# Prevalence of hearing impairment in children at risk

**DOI:** 10.1590/S1808-86942010000600012

**Published:** 2015-10-19

**Authors:** Fernanda Alves Botelho, Maria Cândida Ferrarez Bouzada, Luciana Macedo de Resende, Cynthia Francisca Xavier Silva, Eduardo Araújo Oliveira

**Affiliations:** 1 Master's degree in Health Science - Child and Adolescent Care, Federal University of Minas Gerais (UFMG). Speech therapist at the Clinic Hospital, UFMG; 2 Doctoral degree in Health Science - Child and Adolescent Care, Federal University of Minas Gerais (UFMG). Physician, adjunct professor of the Pediatrics Department, UFMG; 3 Master's degree in speech therapy, Pontifical Catholic University, Sao Paulo. Speech therapist, assistant professor of the Speech Therapy Department, Medical School, UFMG; 4 Undergratuate student, science initiation scholarship at the Medical School, UFMG; 5 Doctoral degree in Health Science - Child and Adolescent Care, Federal University of Minas Gerais (UFMG). Physician, associate professor of the Pediatrics Department, UFMG. Federal University of Minas Gerais - Universidade Federal de Minas Gerais (UFMG)

**Keywords:** audiology, hearing, risk factors, hearing loss, neonatal screening

## Abstract

Hearing impairment is prevalent in the general population; early intervention facilitates proper development.

**Aim:**

To establish the prevalence of hearing impairment in infants at risk, born between June 2006 and July 2008, and to correlate the variables with hearing loss.

**Type of study:**

descriptive and cross-sectional.

**Materials and Methods:**

188 newborns were evaluated using evoked otoacoustic emissions and distortion product and auditory behavior. Tests were repeated if the results were altered. If altered results persisted, the child was referred for impedance testing and, when necessary, for medical evaluation. Infants with normal conduction were referred for brainstem auditory evoked potential testing.

**Results:**

Of 188 children two (1.1%) were excluded, and 174 (92.6%) had results within normal limits. Hearing impairment was found in 12 children (6.3%); hearing loss was retrocochlear in three infants (25%). Unilateral hearing loss was present in two infants (16.7%); bilateral hearing loss was present in 10 infants (83.3%).

**Conclusion:**

The high prevalence of hearing impairment in this population underlines the importance of early audiological testing.

## INTRODUCTION

Impaired hearing is prevalent in the world population. Intervening before the age of 6 months allows for normal development of language regardless of the degree of hearing loss.^1^

This condition may occur in neonates at or not at risk. The prevalence in low-risk neonates ranges from 0.09 to 2.3%;^2^, ^3^ in the high-risk population it ranges from 0.3 to 14.1%.^4^, ^5^ The prevalence of impaired hearing reaches 11% in very low birth weight neonates;^6^ this study, however, included sensorineural, mixed and conduction hearing loss. Only 3% of these subjects were given individual hearing aids. A study that applied electrophysiology methods - evoked otoacoustic emissions - for analyzing neonates found a 6.3% rate of hearing impairment in very low birth weight infants. Another study that evaluated infants in neonatal intensive care units found prevalence rates of hearing loss ranging from 3 to 14.1%.^5^, ^7^

The Joint Committee on Infant Hearing (JCIH) has proposed risk indicators to be used internationally,^8^, ^9^ supplemented by studies to adapt these indicators locally, for instance to the Brazilian context.^10^, ^11^

The purpose of this study was to verify the prevalence of hearing impairment and to correlate it with risk indicators in neonates born and monitored at a tertiary hospital.

## MATERIALS AND METHODS

This descriptive cross-sectional study included neonates weighing 1,500 g or less and a gestational age of not more than 34 weeks, admitted to the neonatal intensive care unit of a reference hospital. After being discharged, these patients were monitored from June 2006 to July 2008. Patients who did not undergo all tests and did not have a final audiological diagnosis during the study period were excluded.

A medical history focused on the risk factors proposed by the JCIH^8^, ^9^ adapted to the Brazilian context.^10^, ^11^ Audiological testing consisted of distortion product evoked otoacoustic emissions (DPOAE) and behavioral observation audiometry (BOA), with reassessments as needed. Other tests, such as immittance testing and brainstem auditory evoked potential (BAEP), were done for diagnostic purposes. Patients were referred to specialists if a diagnosis of hearing loss was made.

A Biologic AUDX 1 device was used for DPOAE testing. Positive results were adequate responses in three of four tested frequencies (5, 4, 3, and 2 kHz).^12^ Testing was done during sleep or in the absence of excessive movement; the microphone of the otoacoustic emissions analyzer was coupled to the outer acoustic meatus with a silicone olive.

Behavioral observation consisted of using non-calibrated instruments (jingle bells and large agogo) to note attention to sounds, exacerbated responses, the cochleopalpebral reflex (CPR), startle, and the habituation phenomenon. Adequate responses were children with the CPR and habituation upon startle.^13^

An Interacoustics AZ7 impedance meter was used in immittance testing; a type A tympanometric curve (Jerger^14^) indicated absence of conductive loss. Acoustic reflexes were evaluated in some children only, those not moving excessively, to support the diagnosis.

A Biologic device with the EP Potentials software was used for BAEP testing; the child was preferably in deep sleep, or else sedated. The stimuli were 21.1 clicks per second, through two registration channels, rarefaction polarity, starting at 90 and 80 dBHL. Silver electrodes on the mastoids, vertex and frontal region picked up the responses. Next, the supra-aural headphones were placed. Wave reproducibility was investigated at stronger intensities, which was then decreased gradually down to the electrophysiological threshold. The manufacturer's (Biologic) reference values were applied as normal values for analyzing wave latency and interpeaks, according to the corrected age of each patient.^15^ BAEP were classified as to the type of response and the type, degree, site and onset of hearing loss.^16^

The response variable was the presence of hearing loss; the independent variables were sex, type of delivery, gestational age, the 1 and 5-minute Apgar scores, weight and whether it was appropriate, small or large for the gestational age, a family history of hearing loss in infancy, congenital infection (toxoplasmosis, rubella, cytomegalovirus (CMV) infection, herpes, and syphilis (TORCH infections), maternal HIV carrier, whether exchange transfusion was made, time in an incubator, and presence and degree of peri-intraventricular hemorrhage, leukomalacia, meningitis, malformation, use of ototoxic drugs, mechanical ventilation, syndromes, use of recreational drugs and/or alcoholic beverages by the mother during pregnancy, and consanguinity of parents.

The Epi Info software was applied for calculating the sample. The assumptions were a 95% confidence interval and a 5% margin of error. The estimated hearing loss for similar populations in the literature was 6.3%.^17^ The calculated sample based on these parameters was 188 patients.

Information gathered from the medical history and audiological testing was entered into a database developed in Epi Info version 2001 (Centers for Disease Control and Prevention, Atlanta, United States of America). The study variables were sex, gestational age, type of delivery, and the presence of risk factors for hearing loss in neonates and lactating infants. Descriptive data resulted from frequencies and percentages of categorical variables, and central tendency measures (mean and median) and measures of dispersion (standard deviation) for quantitative data. The response variable in this study was hearing loss.

Data analysis was a two-step process, starting with the univariate analysis. The chi-square test, Fisher's exact test and the odds ratio were applied for comparing proportions. Student's t test was used for comparing the response variable and the categorical covariates when the usual assumptions of the model were met (normal distribution and homoscedasticity). Otherwise the Mann-Whitney test was applied. The Kolmogorov-Smirnov test was used to check the assumption of normality of the t-test, and the Levene test was applied to check for homocedasticity.^18^ The covariates Apgar score and weight were analyzed categorically and quantitatively. Only one form was included if both were statistically significant. For ease of interpretation, the categorical variable was chosen in such cases.

Multivariate analysis consisted of a logistics regression model, which initially included all variables with a p-value ≤ 0.25 in the univariate analysis - those at least trending towards statistical significance. Next, a stepwise process was applied until only the statistically significant variables (p-value ≤ 0.05) were included in the model; the clinical relevance was also taken into account. The interactions among all covariates in the final regression model were tested. The software R (public domain) was applied for the multivariate analysis.

The institutional review board approved this study (Opinion no. ETIC 271/05). The caretakers of newborn subjects signed a free informed consent form authorizing data gathering.

## RESULTS

The initial sample comprised 188 children of which the medical and family histories were taken. Two patients (1.1%) were excluded - they undertook hearing screening but not BAEP testing by the end of the data-gathering period, and their evaluation was therefore incomplete. Hearing loss was found in 12 infants (6.3%) of the sample. Unilateral hearing loss was detected in two of these infants (16.7%), and bilateral hearing loss was found in the other 10 (83.3%). Among infants with hearing loss, three (25%) had retrocochlear disorders. These children with hearing loss were referred to otorhinolaryngological care and a speech therapy.

The gestational age ranged from 25 to 37 weeks in the 186-subject sample; the median was 31 weeks. The weight ranged from 560 to 2,925 g; the median value was 1,502.5 g. The 1’ Apgar score ranged from 1 to 9 (median - 7) and the 5’ Apgar score ranged from 3 to 10 (median - 9). Table 1 presents the main features of the prenatal period, birth and postnatal period.

**Table 1:** Perinatal factors in the study sample.

Covariate	Frequency	
	n	%
Sex		
Male	94	50,5
Female	92	49,5
Delivery		
Normal	68	36,5
Cesarean section	118	63,5
Weight/gestational age		
AGA	156	83,9
SGA	29	15,6
LGA	1	0,5
Family history of hearing loss		
Yes	15	8,1
No	170	91,4
Unclear	1	0,5
Peri-intraventricular hemorrhage		
Grade I	21	11,3
Grade II	15	8,1
Grade III	14	7,5
None	132	73,1
Weight (grams)		
< 1,000	27	14,5
1,000 to 1,499	66	35,5
≥ 1500	93	50,0

One infant (0.5%) had congenital syphilis, one infant (0.5%) had congenital toxoplasmosis, and another infant (0.5%) had CMV infection. Five mothers (2.7%) were HIV-positive.

Table 2 shows that HIV-positive mothers and use of recreational drugs and/or alcohol by mothers correlated positively with hearing loss in infants.

**Table 2:** Comparison of altered hearing and risk factors for hearing loss in high-risk infants

Covariate	Final result on hearing				p-value	odds ratio	95% CI
	Altered		Normal				
	n	%	n	%			
HIV-positive mother							
Yes	2	16,7	3	1,7	*0,034	11,4	1,2 a 100,4
No	10	83,3	171	98,3		1,0	
Drugs/alcohol use by mother							
Yes	4	33,3	24	13,9	*0,048	4,1	0,9 a 18,3
No	8	66,7	149	86,1		1,0	
Weight (grams)							
< 1,500	9	75,0	84	48,3	0,073	3,2	0,8 a 15,6
≥ 1,500	3	25,0	90	51,7		1,0	
Family history							
Yes	3	25,0	12	6,9	*0,061	4,5	0,8 a 21,8
No	9	75,0	161	93,1		1,0	
Syndrome							
Yes	1	8,3	0	0,0	*0,064		
No	11	91,7	174	100,0		1,0	
Cytomegalovirus infection							
Yes	1	25,0	0	0,0	*0,090		
No	3	75,0	40	100,0		1,0	

Key: HIV (human immunodeficiency virus)

* : Fisher's exact test

Very low birth weight infants tended to have worse results in hearing testing compared to those with higher birth weights. The presence of a family history of hearing loss in infancy increased the chance of an infant presenting hearing loss fivefold; any syndrome also present raised this chance of having hearing loss to 20 times the normal range.

## DISCUSSION

This study describes the prevalence of hearing loss and the features of children born in a tertiary reference hospital and monitored in an outpatient clinic for patients discharged from that hospital. The findings confirm the 6.3% prevalence of hearing loss encountered previously in a survey of very low birth weight infants in Brazil using a similar method (DPOAE and BAEP testing).^17^ Other studies of very low birth weight infants presented lower prevalence values, such as Gill et al. in 1998 (5.56% prevalence), and Roth et al. in 2006 (0.3% rate of sensorineural conditions).^4^, ^19^

Other studies of newborn infants in neonatal intensive care units show higher prevalence rates, which may be the result of multiple risk factors for hearing loss in this group.^5^, ^7^, ^20^

Variation among studies may be due to other prenatal variables, healthcare, and infections that may cause hearing loss. Other factors are the quality of life of a given population, access to adequate nutrition, and the level of culture.

A recent survey in the state of Bahia aimed to investigate the etiology of hearing loss and found that the main cause was rubella of the mother, followed by pyogenic meningitis, idiopathic causes, prematurity, hereditary factors, neonatal jaundice, and others such as chronic otitis media and ototoxicity.^21^ In our data, 0.5% of infants had syphilis, congenital toxoplasmosis or CMV infection.

Congenital toxoplasmosis, which is frequent in Brazil, may cause hearing loss. It may develop subclinically, compromising the early diagnosis of hearing loss. A recent survey in the city of Belo Horizonte showed that the prevalence of congenital toxoplasmosis is 1 for every 1,590 live births. Patients with this disease were referred for audiological investigation, the results of which showed that 21.1% of these infants had sensorineural hearing loss and 10.5% had conduction hearing loss.^22^

Hearing loss in CMV infection is generally progressive. The infant with CMV infection in this study acquired the disease after a blood transfusion; statistical significance, however, was not attained. This is probably because the sample of mothers with prenatal CMV serology is low (only 44 of 186 took this test), resulting in a lesser effect on the data. Additionally, the first signs of hearing loss may present late; thus, a study monitoring the development of hearing and language in children may show a higher prevalence of hearing loss caused by CMV infection.

A systematic review of studies on congenital CMV infection revealed that if the prevalence at birth is 0.7% and the risk of bilateral moderate to profound sensorineural hearing loss is 3 to 5%, the risk of a child developing permanent bilateral hearing loss because of CMV infection is 0.21 to 0.35 per 1,000 births.^23^ A 10-year prospective study on sensorineural hearing loss in children with congenital CMV found a 0.53% prevalence rate in the population. Of those with infection, 5.4% were asymptomatic and 94.6% were symptomatic. Hearing test detected a 22% rate of sensorineural hearing loss. Late onset hearing loss was present in 5% of cases; fluctuating hearing was found in 16% of cases, and progressive hearing loss occurred in 11% of cases. These data showed the need to monitor the hearing of infants with congenital CMV infection.^24^

Our study showed a statistically significant difference in infants of HIV-positive mothers. However, another study of hearing in infants of HIV-positive mothers compared with those of HIV-negative mothers concluded that the risk of sensorineural hearing loss was not higher in the former group.^25^

Other authors have suggested that AIDS should be considered a risk factor for peripheral and/or central hearing loss.^11^ HIV-positive children tended to have hearing disorders of central causes; their acquisition of sound location ability was also compromised.^26^

The study method included DPOAE testing in two or three steps, and immittance and BAEP tests (see Fig. 1). A recent study of 4,519 infants aged not more than 3 years concluded that DPOAE testing (using the same equipment and protocol of the present study) in several steps and monitoring guidelines are useful for detecting hearing loss.^27^

This study started in 2006; at that point, the most recent JCIH recommendations were those of 2000. For completeness, our study included the JCIH indicators of the years 1994 and 2000. New JCIH recommendations were published on October 2007; these guidelines emphasized that high-risk children should be evaluated using BAEP testing because of the risk of auditory neuropathy.^28^ Children with altered initial tests (OAE, BOA) but with no conduction disorders of sound stimuli (immittance testing), and children with normal results in the initial tests but at some risk of central hearing loss (elevated bilirubin levels requiring exchange transfusion, HIV-positive mother, TORCH infections) underwent BAEP testing.

This method enabled the detection of three cases of retrocochlear disorders in the study sample, thereby underlining the concern with such conditions in infants with risk factors. The prevalence of these disorders in children with profound hearing loss was 0.94%;^29^ furthermore, retrocochlear disorders may also occur in children without risk factors.^30^

Monitoring of children aged up to 2 years may also reveal cases of progressive or delayed onset hearing loss, given that the study sample consisted of high-risk infants for all types of hearing loss.

## CONCLUSION

This study aimed to detect the prevalence of auditory disorders in children exposed to several risk factors, because it is thought that the prevalence of hearing loss is high in this population group. In these subjects, multiple factors may result in progressive or delayed onset neonatal or postnatal hearing loss, which should be assessed and monitored in a hearing screening program.

Timely diagnosis and interventions for speech development are not the reality throughout Brazil in most cases of prelingual hearing loss. The population and healthcare professionals involved in childcare should be made aware of the impact of hearing loss; this could result in increased adhesion to neonatal screening programs.
